# Effects of prebiotics on the gut microbiota *in vitro* associated with functional diarrhea in children

**DOI:** 10.3389/fmicb.2023.1233840

**Published:** 2023-09-01

**Authors:** Zhi Du, Jiabin Li, Wei Li, Hao Fu, Jieying Ding, Guofei Ren, Linying Zhou, Xionge Pi, Xiaoli Ye

**Affiliations:** ^1^Department of Pharmacy, Children’s Hospital, Zhejiang University School of Medicine, National Clinical Research Center for Child Health, Hangzhou, Zhejiang, China; ^2^Research Center for Clinical Pharmacy, Zhejiang University, Hangzhou, Zhejiang, China; ^3^Department of Clinical Laboratory, Children’s Hospital, Zhejiang University School of Medicine, National Clinical Research Center for Child Health, Hangzhou, Zhejiang, China; ^4^Institute of Plant Protection and Microbiology, Zhejiang Academy of Agricultural Sciences, Hangzhou, Zhejiang, China; ^5^Department of Pharmacy, Zhejiang Xiaoshan Hospital, Hangzhou, Zhejiang, China; ^6^People's Hospital of Longquan City, Longquan, China; ^7^Department of Medical Administration, Children’s Hospital, Zhejiang University School of Medicine, National Clinical Research Center for Child Health, Hangzhou, Zhejiang, China

**Keywords:** diarrhea, children, prebiotics, fructo-oligosaccharides, gut microbiota

## Abstract

**Purpose:**

Diarrhea is among the top five causes of morbidity and mortality in children. Dysbiosis of the gut microbiota is considered the most important risk factor for diarrhea. Prebiotics have shown efficacy in treating diarrhea by regulating the balance of the gut microbiota *in vivo*.

**Methods:**

In this study, we used an *in vitro* fermentation system to prevent the interference of host-gut microbe interactions during *in vivo* examination and investigated the effect of fructo-oligosaccharides (FOS) on gut microbiota composition and metabolism in 39 pediatric patients with functional diarrhea.

**Results:**

16S rRNA sequencing revealed that FOS significantly improved α- and β-diversity in volunteers with pediatric diarrhea (*p* < 0.05). This improvement manifested as a significant increase (LDA > 2, *p* < 0.05) in probiotic bacteria (e.g., *Bifidobacterium*) and a significant inhibition (LDA > 2, *p* < 0.05) of harmful bacteria (e.g., *Escherichia-Shigella*). Notably, the analysis of bacterial metabolites after FOS treatment showed that the decrease in isobutyric acid, isovaleric acid, NH_3_, and H_2_S levels was positively correlated with the relative abundance of *Lachnoclostridium*. This decrease also showed the greatest negative correlation with the abundance of *Streptococcus*. Random forest analysis and ROC curve validation demonstrated that gut microbiota composition and metabolites were distinct between the FOS treatment and control groups (area under the curve [AUC] > 0.8). Functional prediction using PICRUSt 2 revealed that the FOS-induced alteration of gut microbiota was most likely mediated by effects on starch and sucrose metabolism.

**Conclusion:**

This study is the first to evince that FOS can modulate gut microbial disorders in children with functional diarrhea. Our findings provide a framework for the application of FOS to alleviate functional diarrhea in children and reduce the use of antibiotics for managing functional diarrhea-induced disturbances in the gut microbiota.

## Introduction

Diarrhea is one of the most common diseases among children worldwide (Garbern et al., 2022). Patients with diarrhea exhibit symptoms such as vomiting, fever, watery stools, and abdominal pain. Although the number of diarrhea-related deaths in children has decreased dramatically over time, diarrhea remains one of the five leading causes of morbidity and mortality in this population (Collaborators, 2017; Jin et al., 2018; Reiner et al., 2018). In particular, long-term diarrhea due to functional diarrhea may cause electrolyte disturbances in the body, affecting the absorption of nutrients. If functional diarrhea is not treated in a timely manner, it can affect the child’s growth and development and even become life-threatening (Burgers et al., 2020; Negesse et al., 2022). The improved management of functional diarrhea as a preventable diarrhea requires a targeted approach and more effective prevention and treatment interventions.

Many studies have also documented changes in the gut microbiota before, during and after diarrheal episodes (Hsiao et al., 2014; Youmans et al., 2015). Moreover, a case–control report from the Global Enteric Multicenter Study revealed that moderate-to-severe pediatric diarrhea alters the microbiota composition and decreases bacterial diversity (Pop et al., 2014). Children experience multiple episodes of diarrhea due to the defective development of the gut microbiota, which can lead to functional diarrhea (Shin et al., 2019; Rouhani et al., 2020). Therefore, modulation of the gut microbiota could be effective in preventing functional diarrhea.

Probiotics are non-pathogenic live microbes that can reverse gut dysbiosis by improving the microbial balance in the gut. Since probiotics are typically non-pathogenic bacteria that already exist in the human gut, they are often considered harmless. Probiotics have been shown to be effective in treating diarrhea caused by multiple factors, including traveler’s diarrhea, acute rotavirus diarrhea, diarrhea due to radiation therapy, and diarrhea due to lactose intolerance (Hill et al., 2014; Rondanelli et al., 2017; Sanders et al., 2019). Studies have also shown that probiotics can prevent bacterial translocation, change the composition of the gut microbiota, preserve the integrity of the gut barrier, and alter the local immunological response of the gut-associated immune system (Mantegazza et al., 2018).

Prebiotics are recognized as “substrates that are selectively used by probiotics and produce beneficial effects” (Gibson et al., 2017). The intake of prebiotics can promote probiotic colonization and increase short-chain fatty acid (SCFA) production, which promotes the maintenance of intestinal barrier integrity (Azad et al., 2020; Snelson et al., 2021). By shortening the duration of acute watery diarrhea, several prebiotics — including fructo-oligosaccharides (FOS), inulin, and pectin oligosaccharides — can effectively treat diarrhea (Rigo-Adrover et al., 2017; Pujari and Banerjee, 2021). However, most studies on prebiotics have been conducted in animals and adults with diarrhea. Meanwhile, studies on children with diarrhea are relatively limited. Moreover, *in vivo* studies on prebiotics can be confounded by host–microbe interactions in the gut. Therefore, in this study, we adopted an *in vitro* fermentation model to study the direct effect of prebiotics on the gut microbiota in children with functional diarrhea.

## Materials and methods

### Chemicals

FOS (purity ≥95% and moisture ≤5%) was purchased from Shandong Baoling Bao Biological Co. Ltd., China. Tryptone, bile salt, yeast extract, L-cysteine, and heme were purchased from Sigma Company, USA. Phosphate-buffered saline (PBS), NaCl, KH_2_PO_4_, K_2_HPO_4_, MgSO_4_, CaCl_2_, metaphosphoric acid, and crotonic acid were purchased from Sangon Biotech (Shanghai) Co., Ltd., China.

### Collection of fresh fecal samples from children with functional diarrhea

The study included 39 child participants (aged 6–15 years) with functional diarrhea diagnosed based on the Rome IV Criteria who had been living in Hangzhou, Zhejiang Province, China. All participants had experienced recurrent discomfort or abdominal pain associated with no less than two of the following features for at least 1 day per week in the last 3 months: (1) pain related to defecation; (2) onset related to a change in the frequency of feces; and (3) the immunocompetent patient with “watery” diarrhea of at least 4 weeks duration. Further details about inclusion criteria were as described previously (Smalley et al., 2019). Children with illnesses other than functional diarrhea and those treated with antibiotics, prebiotics, or probiotics within the previous month were excluded. Fresh fecal samples were collected from volunteers with pediatric functional diarrhea in a 30 mL sterile stool sample box (91 mm × 24 mm; BioRise Co., Ltd., China). At least 4 g of partially processed feces with low amounts of undigested food residue and minimal contact with air after defecation were transferred to an anaerobic jar and stored at 4°C. All samples were tested within 4 h. The study was approved by the Ethical Committee of Hangzhou Centers for Disease Control and Prevention (No. 202047).

### Isolation of gut microbiota

Three 1.5 mL sterile centrifuge tubes were each filled with 0.2 g of fresh feces and stored in a refrigerator at −80°C as backup original fecal samples. Subsequently, 8 mL of sterile PBS and 0.8 g of fresh fecal samples were added to 10 mL sterile centrifuge tubes. The tubes were sealed with tape and placed on a shaker for thorough mixing. The supernatant was collected after filtration to obtain a 10% gut microbiota extraction solution.

### *In vitro* anaerobic fermentation by the gut microbiota

The isolated gut microbes were inoculated into a simulated gut fermentation system and incubated at 37°C for 24 h under anaerobic and airtight conditions, as described previously (Liu et al., 2020). The Yeast Casitone Fatty Acids (YCFA) medium contained the following (per 100 mL): 4.5 g/L yeast isolate, 3.0 g/L tryptone, 3.0 g/L peptone, 0.4 g/L bile salt, 0.8 g/L cysteine hydrochloride, 4.5 g/L NaCl, 2.5 g/L KCl, 0.45 g/L MgCl_2_, 0.2 g/L CaCl_2_, 0.4 g/L KH_2_PO_4_, 1.0 mL Tween 80, 1.0 mL resazurin, and 2.0 mL of a trace element solution. After sufficient dissolution and boiling, 4.5 mL of the YCFA medium was injected into a nitrogen-filled cilium vial. Finally, the vials were fixed with caps and sterilized using high-pressure steam. FOS were added to the YCFA medium (0.8 g/100 mL) to obtain the FOS treatment group for diarrhea (FOSD), as the mod. Pure YCFA medium was used to obtain the control treatment group for diarrhea (CtrlD).

### 16S rRNA high-throughput sequencing of gut microbiota

Genomic DNA was extracted from the gut microbiota using the FastDNA® Spin Kit for Soil (MP Biomedicals, United States) based on the manufacturer’s instruction. Using a thermocycler PCR system (GeneAmp 9,700, ABI, San Diego, CA, United States), the V3–V4 hypervariable regions of the bacterial 16S rRNA gene were amplified with the primers 341F (5′-CCTAYGGGRBGCASCAG-3′) and 806R (5′-GGACTACHVGGGTWTCTAAT-3′). The PCR conditions were as described previously (Pi et al., 2022). Purified amplicons were pooled in equimolar amounts, and paired-end sequencing was performed on the NovaSeq PE250 platform (Illumina, San Diego, United States) based on the standard protocol of Majorbio Bio-Pharm Technology Co. Ltd. (Shanghai, China).

Amplicon sequence variants (ASVs) were identified by denoising the streamlined sequences after quality control splicing using the DADA2 plug-in in QIIME2 (version 2020.2). The Naive Bayes agreement taxonomy classifier in QIIME2 and the SILVA 16S rRNA database (v138) were used to analyze the taxonomic profiles of the ASVs. Protocol sequence data for all original fecal samples and all fermentation samples were submitted to the National Center for Biotechnology Information Short Read Archive under the serial no. PRJNA978990.

### Determination of SCFAs during *in vitro* fermentation

After 24 h of *in vitro* fermentation, the composition and content of SCFAs were analyzed. The contents of six SCFAs — including acetic acid (Ace), propionic acid (Pro), isobutyric acid (Isob), butyric acid (But), isovaleric acid (Isov), and valeric acid (Pen) — were determined. Significant age-related differences in total SCFAs and in each type of SCFA were analyzed. Heat maps and Spearman correlation coefficients were used to analyze the correlation between SCFAs showing significant variation and the relative abundance of bacterial genera.

To identify SCFAs, 2.5 g of metaphosphoric acid was dissolved in 100 mL of deionized water, and 0.6464 g of crotonic acid was added to obtain a crotonic acid/metaphosphoric acid solution. The fermentation solution (500 μL) was mixed well with the crotonic acid/metaphosphoric acid solution (100 μL), placed at −40°C for 24 h, and then centrifuged at 4°C and 13,000 r/min for 3 min. Finally, the supernatant was filtered using a 0.22-μm microporous membrane. A gas chromatograph was loaded with the sample. Subsequently, the aging strategy was adopted. The gas chromatography conditions were as described previously (Pi et al., 2022). The acquired data were recorded.

### Determination of gases during *in vitro* fermentation

Fermentation-completed bottles were removed and cooled to 25°C. Subsequently, gas detection was performed using a gut microbial gas detector according to a previously described method (Ye et al., 2022). After the gas detector was turned on and preheating was completed, the record button was pressed, and the inlet and outlet ports were connected to the syringe bottle through a rubber tube and a disposable syringe needle, respectively. The needle was not allowed to touch the liquid surface of the culture medium during the measurement to prevent water ingress into the machine. The machine detected the concentration of CH_4_, H_2_S, NH_3_, CO_2_, and H_2_. The concentration of the five gases increased and then decreased over time, with the highest value representing the volumetric concentration of the gas produced by gut microbiota fermentation. After the values of all five gases had dropped to zero, the next sample bottle was tested.

### Statistical analysis

Microbial data were analyzed on the online Majorbio Cloud Platform.^1^ Gas and SCFA data were plotted using GraphPad Prism 8.0.3 (GraphPad Software Inc., United States) and statistically analyzed with SPSS 23 (IBM Crop., United States). All results are presented as the means ± SEM (80 independent experiments × 3 parallel experiments). The distribution characteristics of all data were tested using the Shapiro–Wilk test. For multiple group comparisons, the Friedman test was performed for data with non-normal distribution, and one-way ANOVA and Tukey’s comparison tests were performed for data with normal distribution. For two-group comparisons, the Wilcoxon rank-sum test was performed for data with non-normal distribution, and the parametric t-test was performed for data with normal distribution. Statistical significance thresholds were as follows: *0.01 < *p* ≤ 0.05; **0.001 < *p* ≤ 0.01; and ***p ≤ 0.001.

## Results

### Alpha-diversity and beta-diversity

**Figure 1:**
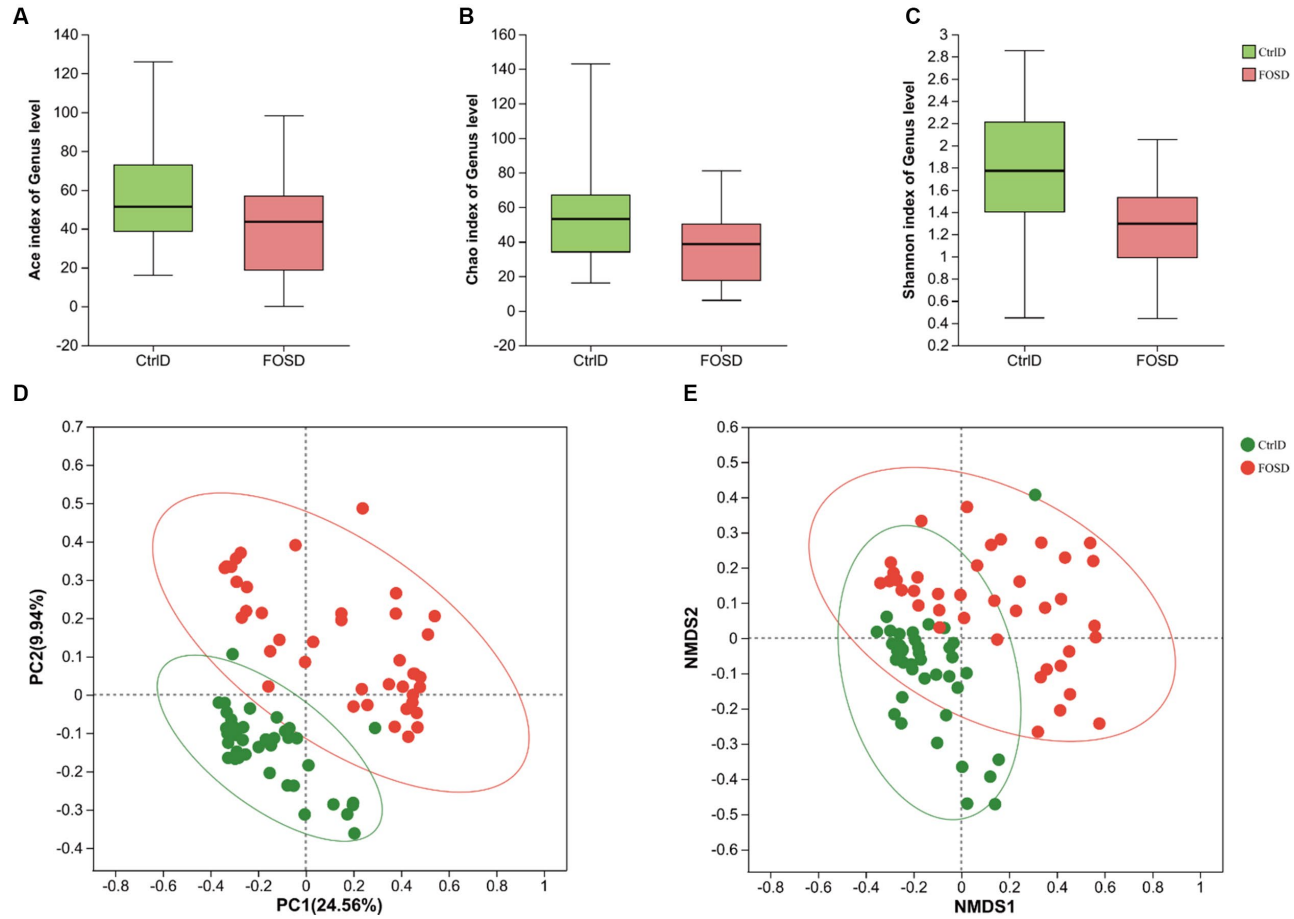
Diversity of gut microbiota after *in vitro* fermentation. α-diversity in the FOS and YCFA groups based on the **(A)** ACE index (*p* = 0.008715), **(B)** Chao index (*p* = 0.001438), and **(C)** Shannon index (*p* = 0.000206) examined using the Wilcoxon rank-sum test. β-diversity at the genus level in the FOS and YCFA groups based on **(D)** PCoA (*p* = 0.001) and **(E)** NMDS analysis (*p* = 0.001).

After *in vitro* fermentation, various gut microbes were examined using 16S rRNA sequencing. Figures 1A–C show the α-diversity in the CtrlD and FOSD groups. The ACE (*p* = 0.008715), Chao (*p* = 0.001438), and Shannon (*p* = 0.001438) indices differed significantly between the two groups (*p* < 0.05), indicating a significant difference in the community-level diversity between the FOSD and CtrlD groups. Figures 1D,E show the β-diversity of the FOSD and CtrlD groups. PCoA (*p* = 0.001) and NMDS analysis (*p* = 0.001) revealed a significant difference in the genus-level structure of the bacterial community between the FOSD and CtrlD groups during *in vitro* fermentation (Figures 1D,E).

### Microbiome analysis

**Figure 2:**
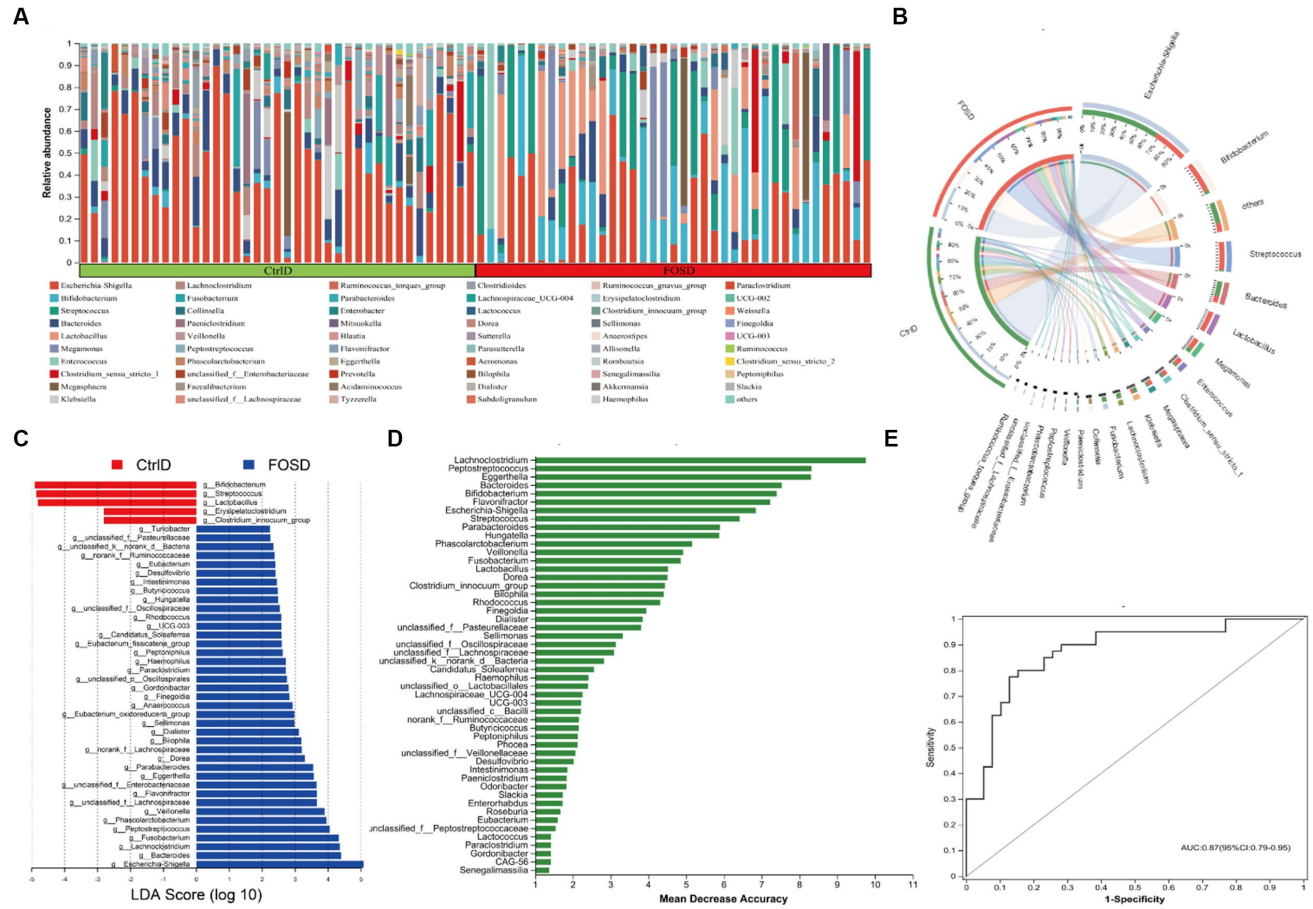
Composition of gut microbiota after *in vitro* fermentation: **(A)** Community bar plot analysis at the genus level shows the relative abundance of fecal microbes in each sample from the FOSD and CtrlD groups. **(B)** Circos analysis at the genus level displays the abundance relationship between samples and bacterial communities. **(C)** Gut microbiota comparisons at the genus level between the FOSD and CtrlD groups analyzed using LEfSe (LDA > 2, *p* < 0.05); **(D)** Bar plot showing the variable importance of gut microbes at the genus level constructed using random forest model analysis. **(E)** Performance of the model candidates assessed using the ROC analysis of gut microbes at the genus level: AUC = <0.5, no diagnostic value; AUC = 0.5–0.7, low accuracy; AUC = 0.7–0.9, certain degree of accuracy; AUC = >0.9, high accuracy.

The relative abundance of different genera differed among the 79 fecal samples, as shown by the bar plots in Figure 2A. Visualization of the corresponding abundance relationships between the samples and bacterial communities at the genus level using Circos analysis confirmed the results of the bar plot analysis (Figure 2B). Figure 2B shows the average relative abundance of the five genera predominant in fecal samples from both the FOSD and CtrlD groups, including *Escherichia-Shigella* (19.09% vs. 43.26%), *Bifidobacterium* (18.13% vs. 1.80%), *Streptococcus* (16.48% vs. 8.44%)*, Bacteroides* (4.19% vs. 8.96%), and *Lactobacillus* (12.66% vs. 1.04%), respectively. To further determine whether specific bacterial taxa were differentially enriched in the FOSD group versus the CtrlD group, we applied the LEfSe analysis method, which is based on the LDA binding effect size. As shown in Figures 2C, 44 genera exhibited differences in content between the FOSD and CtrlD groups. For example, a significant enrichment of *Bifidobacterium*, *Streptococcus*, *Lactobacillus*, and *Erysipelatoclostridium* was identified in the FOSD group. Meanwhile, *Escherichia-Shigella*, *Bacteroides*, *Lachnoclostridium*, and *Fusobacterium* were significantly more abundant in fecal samples from the CtrlD group. We further evaluated the value of the gut microbiota as a biomarker using random forest model analysis. The AUC-Random Forest algorithm was used to identify an optimal random forest model that maximizes the AUC value of the ROC curve. In the validation queues of the FOSD and CtrlD groups, the first 18 genera were selected in order of feature importance to distinguish the FOSD group from the CtrlD group, as shown in Figure 2D. We conducted specificity and sensitivity analyses for the first 18 genera. The AUC was 0.87 (95% confidence interval [CI]: 0.79–0.95) (Figure 2E).

**Figure 3:**
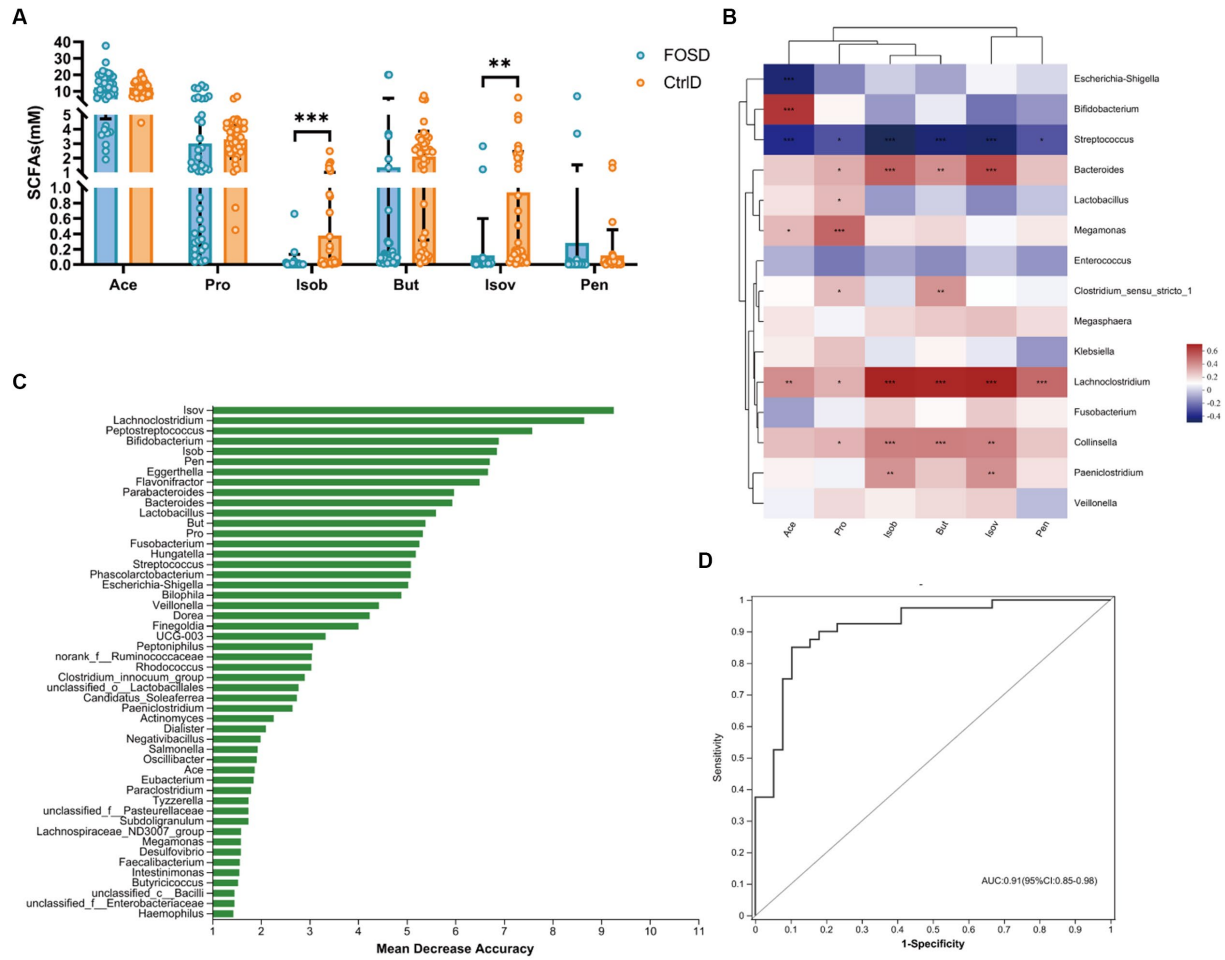
Content of gas in different media during *in vitro* fermentation. **(A)** Levels of five gases in the FOSD and CtrlD groups. **(B)** Spearman correlation heatmap between gut microbes (genus level) and gases. **(C)** Bar plot showing the variable importance of gut microbes at the genus level and gases established using random forest model analysis; **(D)** Performance of model candidates assessed using the ROC analysis of gut microbes at the genus level and gases: AUC = <0.5, no diagnostic value; AUC = 0.5–0.7, low accuracy; AUC = 0.7–0.9, certain degree of accuracy; AUC = >0.9, high accuracy. Statistical significance thresholds: * 0.01 < *p* ≤ 0.05; ** 0.001 < *p* ≤ 0.01; *** *p* ≤ 0.001.

### SCFA analysis

**Figure 4:**
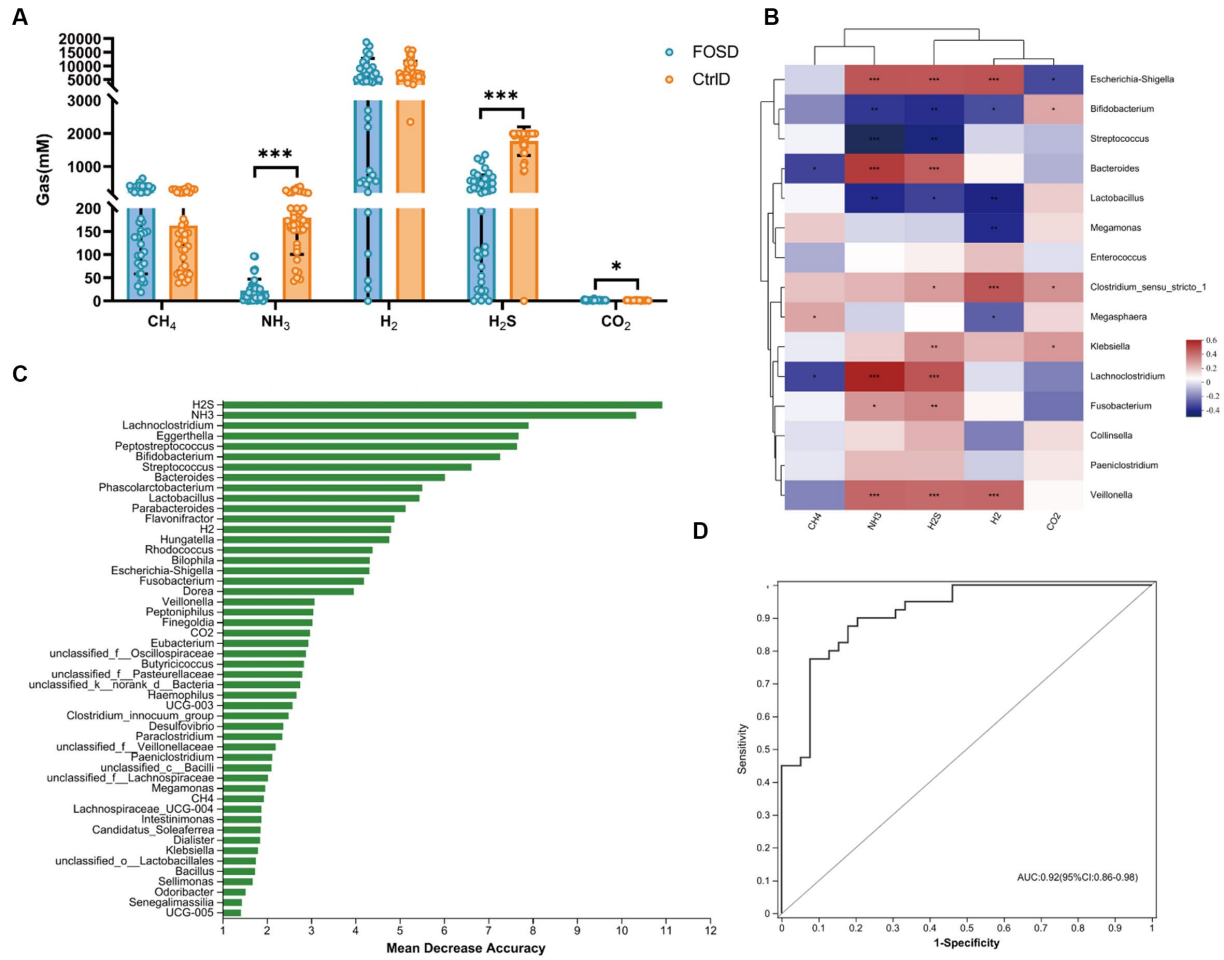
SCFA levels in the FOSD and CtrlD groups during *in vitro* fermentation. **(A)** Levels of six SCFAs in the FOSD and CtrlD groups. **(B)** Spearman correlation heatmap between gut microbes (genus level) and SCFAs; **(C)** Bar plot showing the variable importance of gut microbes at the genus level and SCFAs established using random forest model analysis; **(D)** Performance of model candidates assessed using the ROC analysis of gut microbes at the genus level and SCFAs: AUC = <0.5, no diagnostic value; AUC = 0.5–0.7, low accuracy; AUC = 0.7–0.9, certain degree of accuracy; AUC = >0.9, high accuracy. Statistical significance thresholds: * 0.01 < *p* ≤ 0.05; ** 0.001 < *p* ≤ 0.01; *** *p* ≤ 0.001.

The composition and content of SCFAs were analyzed after 24 h of *in vitro* fermentation. Six SCFAs, including Ace, Pro, Isob, But, Isov, and Pen, were evaluated (Figure 4A). Compared to the CtrlD group, the FOSD group showed significantly lower levels of Isob (*p* ≤ 0.001) and Isov (*p* ≤ 0.01) (Figure 4A). Random forest model analysis revealed that among all the SCFAs tested, Isob and Isov were the most valuable biomarkers for distinguishing between the FOSD and CtrlD groups (Figure 4B). ROC curves of the gut microbiota and SCFAs were used to evaluate their sensitivity and specificity in distinguishing between the FOSD and CtrlD group, and the AUC was found to be 0.91 (95% CI: 0.85–0.98), as shown in Figure 4C. We used heatmaps and Spearman correlation coefficients to analyze the association between SCFAs showing significant changes and the relative abundance of bacterial genera (Figure 4D). Isob and Isov were positively correlated with *Lachnoclostridium*, *Bacteroides*, *Collinsella*, and *Paeniclostridium* but showed a significant negative correlation with *Streptococcus*.

### Gas analysis

The composition and content of gases were analyzed after 24 h of *in vitro* fermentation. H_2_S (*p* ≤ 0.001) and NH_3_ (*p* ≤ 0.001) levels were significantly lower in the FOSD group than in the CtrlD group (Figure 3A). Using random forest model analysis, we identified H_2_S and NH_3_ as the most valuable microbial gas biomarkers for distinguishing between the FOSD and CtrlD groups, as shown in Figure 3B. ROC curves of the gut microbiota and SCFAs were used to evaluate their sensitivity and specificity in distinguishing the FOSD group from the CtrlD group, and the AUC was 0.92 (95% CI: 0.85–0.98), as shown in Figure 3C. To analyze the relationships between gases and the gut microbiota, Spearman correlation heatmaps were adopted (Figure 3D). Among the eight bacterial genera with the highest differences in abundance, *Escherichia-Shigella*, *Bacteroides*, *Lachnoclostridium*, and *Veillonella* were positively correlated with H_2_S and NH_3_ levels, whereas *Bifidobacterium*, *Streptococcus*, and *Lactobacillus* showed a significant negative correlation.

### Functional prediction

**Figure 5:**
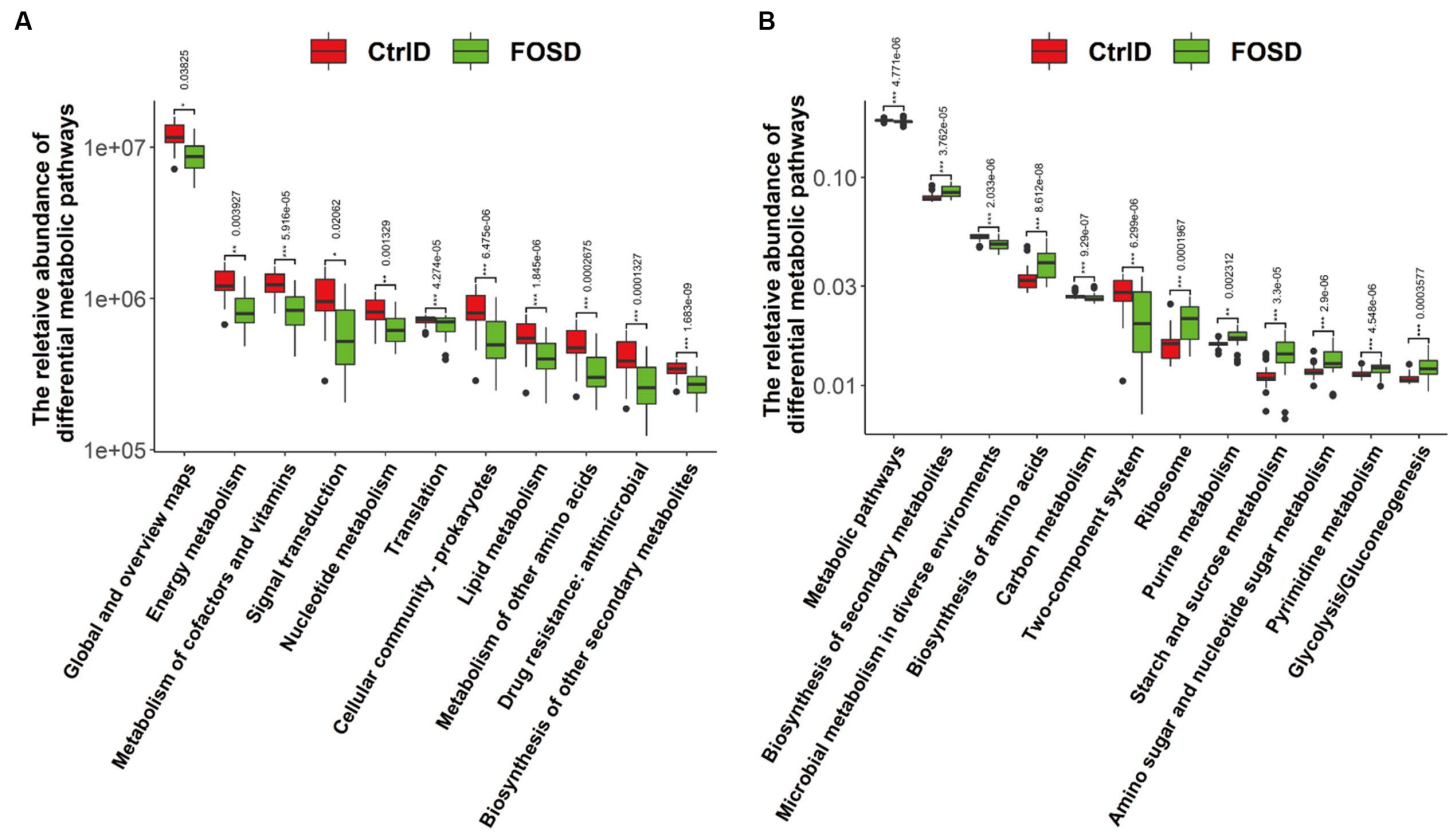
PICRUSt 2 functional prediction and Wilcoxon rank-sum test bar plots. Abundance of metabolic pathways based on KEGG categories at Level 2 **(A)** and Level 3 **(B)**.

In order to predict and compare gut microbiota function between the FOSD and CtrlD groups, PICRUSt analysis for functional prediction was performed based on the KEGG database. Further, STAMP software was used to analyze the prediction results (*p* < 0.05), and pathways with a proportion of average relative abundance <1% were screened out (Figure 5). At Level 2, 12 metabolic pathways were found to be significantly different (Figure 5A). Among them, six metabolic pathways were significantly more enriched in the FOSD group than in the CtrlD group, including global and overview maps (*p* = 0.006762) and nucleotide metabolism (*p* = 1.845e^−6^). Moreover, six metabolic pathways were significantly more enriched in the CtrlD group than in the FOSD group, including energy metabolism (*p* = 1.683e^−9^) and metabolism of cofactors and vitamins (*p* = 0.0001327). At Level 3, 12 metabolic pathways were found to be significantly different (Figure 5B). Among them, eight were significantly more enriched in the FOSD group, including biosynthesis of secondary metabolites (*p* = 4.548e^−6^) and biosynthesis of amino acids ribosome (*p* = 3.3e^−5^), while four were significantly more enriched in the CtrlD group, including metabolic pathways (*p* = 0.0003577) and microbial metabolism in diverse environments (*p* = 2.9e^−6^).

## Discussion

The communication between the gut microbiota and prebiotics could uncover the mechanistic role of the microbiota and its metabolic actions in the pathophysiology of pediatric diarrhea. This study aimed to obtain an improved understanding of the gut dysbiosis induced by functional diarrhea in childhood and the ameliorative effect of prebiotics on this condition.

Comprehensive information on the structural and functional configuration of the gut microbiota in school-age children is limited. In this study, we obtained a comprehensive overview of the gut microbial characteristics of 39 school-age children with functional diarrhea following *in vitro* fermentation. Our findings revealed that the microbiomes of the FOSD group (the model of children with functional diarrhea treated by FOS) exhibited increased microbial α- and β-diversity than those of the CtrlD group (the model of children with functional diarrhea). Diversity of the gut microbiota is essential for maintaining stable gut function (Liu et al., 2022). Prebiotics, such as FOS, inulin, and pectic oligosaccharides, act as soluble decoy receptors that mimic the binding sites of pathogens, inhibiting pathogen colonization and facilitating pathogen elimination from the gut (Pujari and Banerjee, 2021). Some studies have shown that prebiotics can exert a significant therapeutic effect and shorten the duration of acute watery diarrhea (Li et al., 2021a). Therefore, enhancement of gut microbial diversity is likely to underlie the FOS-induced defense against pediatric functional diarrhea.

Accumulating evidence has demonstrated that the gut microbiota maintains normal gut function, and an improvement in the gut’s microbial structure facilitates the amelioration of diarrhea (Palm et al., 2015; Song et al., 2016; Liu et al., 2018; Li et al., 2021b). We compared gut microbiota composition between the FOSD and CtrlD groups and found that FOS remarkably increases the abundance of *Bifidobacterium*, *Streptococcus*, *Lactobacillus*, etc., while significantly reducing the abundance of *Escherichia-Shigella*, *Bacteroides*, *Lachnoclostridium*, *Fusobacterium*, etc. Characterization of the gut microbiota has shown that the relative abundance of *Lactobacillus* and *Bifidobacterium* is significantly lower in patients with functional diarrhea than in healthy controls (Negesse et al., 2022). The mixture of *Bifidobacterium*, *Streptococcus*, and *Lactobacillus* in probiotic blends significantly relieves abdominal pain in patients with moderate-to-severe diarrhea (Skrzydło-Radomańska et al., 2021). Limiting the growth of these harmful bacteria (*Escherichia-Shigella*, *Lachnoclostridium*, *Fusobacterium*, etc.) in the gut can also improve diarrhea symptoms (Ma et al., 2020; Hong et al., 2023). Using random forest analysis, we identified specific gut microbial markers for differentiating the FOSD group from the CtrlD group. ROC curves for distinguishing between the FOSD group and the CtrlD group based on the gut microbes showed high accuracy. These findings suggest that characterization based on probiotics and harmful bacteria in the gut may be useful for analyzing the effects of FOS interventions in children with functional diarrhea.

Alterations in microbial metabolites following diarrhea in children have been linked to changes in the gut microbiota (Rhoades et al., 2021). SCFAs are important fermentation products generated by the gut microbiota and can affect the integrity of the intestinal mucosa, glycolipid metabolism, and immune and inflammatory responses. They have also been implicated in the pathogenesis of functional diarrhea (Xiao et al., 2021). Ace, Pro, and But — which are produced from carbohydrates in the colon and are correlated with the gut microbiota — account for 95% of all gut SCFAs (Rajilić-Stojanović et al., 2015). In our study, the levels of Ace, Pro, and But did not differ between the FOSD and CtrlD groups. However, the levels of Isob and Isov showed a significant decrease in the FOSD group. Isob and Isov have received extremely little attention due to their very low levels in the gut (Xiao et al., 2021). However, several studies have reported that high and low concentrations of SCFAs produce facilitative and inhibitory effects, respectively, on accelerated transit rates in patients with functional diarrhea (Treem et al., 1996; Tana et al., 2010). Therefore, the significant reduction in Isob and Isov induced by FOS is likely to improve the diarrheal symptom of an accelerated transit rate in children. Further analysis in our study revealed that this effect of FOS may be closely related to the reduced relative abundance of *Lachnoclostridium*, and that Isob, Isov, and *Lachnoclostridium* are important biomarkers of FOS-induced improvement in childhood functional diarrhea.

In addition to SCFAs, gases are also important metabolites produced by the gut microbiota. The main gases produced by the gut microbiota are CH_4_, H_2_, H_2_S, NH_3_, and CO_2_ (Louis and Flint, 2017). In our study, the levels of H_2_S and NH_3_ were found to be significantly lower in the FOSD group, indicating that FOS can inhibit the production of H_2_S and NH_3_. Interestingly, evidence shows that the levels of H_2_S and NH_3_ are closely related to the severity of diarrhea (Banik et al., 2016; Chojnacki et al., 2022). H_2_S as a signaling molecule is involved in several pathological processes in the gut, including inflammation and mucosal damage, and it is a risk factor for diarrhea-related diseases such as colitis and colon cancer (Villanueva-Millan et al., 2022). Meanwhile, the excess production of NH_3_ is responsible for increased intestinal secretion and contractions, which lead to diarrhea (Chojnacki et al., 2022). Therefore, the reduction in H_2_S and NH_3_ production due to FOS could be beneficial in improving the symptoms associated with diarrhea in children. Notably, our results also showed that H_2_S and NH_3_ could represent another important class of biomarkers for monitoring the FOS-induced improvement in pediatric functional diarrhea.

PICRUSt 2 analyses (level 3) in this study revealed that FOS alters bacterial metabolic pathways in childhood functional diarrhea. Starch and sucrose metabolism (*p* = 8.612e^−8^) were the most affected by FOS. Undigested starch and sucrose promote bacterial fermentation and gas production, with water diffusion, causing abdominal bloating, pain, and diarrhea. Probiotics such as *Bifidobacterium* and *Lactobacillus* can significantly relieve diarrhea by increasing starch and sucrose metabolism (Li et al., 2022; Bai et al., 2023). Therefore, it is highly likely that the promotion of *Bifidobacterium* and *Lactobacillus* proliferation by FOS led to improvements in starch and sucrose metabolism, facilitating recovery from functional diarrhea among children.

We acknowledge that the present study has several limitations. First, the findings *in vitro* fermentation system cannot fully reflect the intestinal fermentation in the human body. Second, the effects of gases on health remain poorly studied, and tools and techniques to accurately quantify the different gases produced in the gut are unavailable. Although we measured the production of various gases through our *in vitro* simulated fermentation system, how these gases — including H_2_S and NH_3_ — regulate the gut microbiota and influence metabolism to promote human health remains to be explored. Furthermore, 16S rRNA sequencing technology cannot provide information on the functional characteristics of the gut microbiota. Hence, we could not evaluate the metabolic pathways contributing to the synthesis of FOS-related metabolites. Techniques such as metagenomics can be applied to better understand the functional characteristics of the gut microbiota in pediatric patients with functional diarrhea. Finally, more experimental techniques and analytical methods need to be adopted to explore the regulatory effect of FOS on the gut microbiota associated with functional diarrhea in children.

## Conclusion

Diarrhea is among the most significant contributors to poor health and mortality in children. Functional diarrhea is caused by a disturbance in gut microbiota, especially the decline of microbial diversity, predominance of harmful bacteria, and alteration of bacterial metabolites and metabolic pathways. FOS, a plant-derived prebiotic, can significantly increase the diversity of gut microbiota in children with functional diarrhea, promote the multiplication of probiotic bacteria, and inhibit the generation of harmful bacterial metabolites, thus facilitating recovery from functional diarrhea. The relevant differential metabolic pathways identified by microbial functional prediction analysis in this study could help us uncover the exact mechanism underlying the FOS-induced regulation of the gut microbiota for functional diarrhea management in children. Our findings provide innovative insights into the effects of FOS in the regulation of gut microbes during non-infectious pediatric diarrhea and indicate that FOS could be a powerful alternative to antibiotics, potentially reducing their clinical application.

## Data availability statement

The datasets presented in this study can be found in online repositories. The names of the repository/repositories and accession number(s) can be found below: https://www.ncbi.nlm.nih.gov/, PRJNA978990.

## Ethics statement

The studies involving humans were approved by the Ethical Committee of Hangzhou Centers for Disease Control and Prevention (No. 202047). The studies were conducted in accordance with the local legislation and institutional requirements. The human samples used in this study were acquired from The human samples used in this study were acquired from fresh fecal. Written informed consent for participation was not required from the participants or the participants’ legal guardians/next of kin in accordance with the national legislation and institutional requirements.

## Author contributions

ZD: funding acquisition, data curation, formal analysis, and writing – original draft. JL: data curation, formal analysis, and methodology. WL and HF: data curation and formal analysis. JD, GR, and LZ: sample collection. XP: methodology, supervision, project administration, and review. XY: methodology, supervision, project administration, and review. All authors contributed to the article and approved the submitted version.

## Funding

This research was supported by the Hangzhou Agricultural and Society Development Project (202004A20) and the Natural Science Foundation of Zhejiang Province (LYY22H280002).

## Conflict of interest

The authors declare that the research was conducted in the absence of any commercial or financial relationships that could be construed as a potential conflict of interest.

## Publisher’s note

All claims expressed in this article are solely those of the authors and do not necessarily represent those of their affiliated organizations, or those of the publisher, the editors and the reviewers. Any product that may be evaluated in this article, or claim that may be made by its manufacturer, is not guaranteed or endorsed by the publisher.

## Footnotes

^1^ https://www.majorbio.com
